# Paradoxical Increase in Body Mass Induced by Beta-Guanidinopropionic Acid in Juvenile Spontaneously Hypertensive Rats

**DOI:** 10.7759/cureus.19394

**Published:** 2021-11-09

**Authors:** L M Brewster

**Affiliations:** 1 Cardiovascular Disease and Cardiovascular Population Health, Creatine Kinase Foundation, Amsterdam, NLD

**Keywords:** beta-guanidinopropionic acid, blood pressure, creatine kinase, gaba, paradoxical growth, spontaneously hypertensive rat

## Abstract

Background

The adenosine triphosphate (ATP) regenerating enzyme creatine kinase (CK) is intimately involved in blood pressure generation. Consequently, the creatine transporter and CK inhibitor beta-guanidinopropionic acid (GPA) successfully reduced blood pressure in 16-week-old spontaneously hypertensive rats (SHR), but GPA may cause growth retardation in juvenile mammals. This report considers a serendipity observation of paradoxical growth increase after using GPA to prevent hypertension in three-week-old SHR.

Methods

Implementing the “Animal Research: Reporting of In Vivo Experiments” (ARRIVE) guideline, male, three-week-old spontaneously hypertensive rats (N=22) were randomly assigned to standard soy-based (creatine-free) chow with GPA 0.1% vs control chow during four weeks (primary, t=4w) or six weeks of treatment (t=6w). Blood pressure measured by the tail-cuff method was the main outcome. Other outcomes included body mass and contractility characteristics of isolated arteries.

Results

Body mass at baseline was 28.4 (SE 0.71) g (n=22). With similar food intake/100 gram animal in both groups, GPA-treated rats (n=11) developed a strikingly larger body size and mass: t=4w, GPA 110.4 g (3.7) vs controls (n=11) 65.0 g (4.8) (+69.8%; p<0.001); t=6w, GPA 154.3 (4.7) vs controls 68.0 (4.7) g. There were no significant differences in cardiovascular parameters including blood pressure.

Discussion

An unexpected increase in body mass and size without concurrent blood pressure increase was observed in juvenile SHR on GPA vs control soy-based chow. It is speculated that the partial creatine agonist activity of GPA contributed to these effects. Further studies are needed to confirm these findings and better understand the impact of modulating energy metabolism in juvenile hypertension-prone mammals.

## Introduction

Beta-guanidinopropionic acid (or N-(aminoiminomethyl)-beta-alanine; C_4_H_9_N_3_O_2_, GPA) is a structural isomer and competitive inhibitor of creatine (C_4_H_9_N_3_O_2_) [1-3]. GPA is used in experimental settings to inhibit the creatine kinase enzyme system (CK, EC 2.7.3.2) [1-3]. The enzyme catalyzes the reaction [1]:

Phosphocreatine + MgADP + H^+^ ↔ Creatine + MgATP

CK is ubiquitously present in cytoplasm and mitochondria, with high activity levels found in tissues with high and fluctuating energy demands. ATP generated by F1FoATP synthase in the mitochondrial matrix is transported by adenine nucleotide translocase to the mitochondrial intermembrane space, where mitochondrial CK catalyzes the formation of phosphocreatine from ATP. Subsequently, phosphocreatine is transported to the cytosol via the outer membrane voltage-dependent anion channel. Cytosolic CK is tightly bound near ATPases, where it utilizes phosphocreatine to rapidly resynthesize ATP, including plasma membrane Na^+^/K^+^-ATPase, sarco/endoplasmic reticulum membrane Ca^2+^-ATPase, and myosin ATPase at the M-line of myofibrils. Thus, the intracellular CK system functions as a dynamic, temporal, and spatial energy buffer that may greatly enhance muscle contractility and ion transport. Other metabolic functions of this central regulatory enzyme of energy metabolism include proton buffering, and the indirect regulation of glycogenolysis, glycolysis, insulin resistance, and mitochondrial activity. CK is central to growth and development, and its activity is associated with insulin resistance and obesity in adults [1-5]. In recent years, it was recognized that creatine kinase facilitates the contractility of vascular and cardiac tissue and renal sodium retention [1,4-5]. CK was strongly associated with blood pressure, up to 20 mmHg higher systolic blood pressure (SBP)/log CK increase in humans [1,4-5]. GPA, acting as a creatine transporter inhibitor and intracellular CK inhibitor, has been used to inhibit CK, leading to lower blood pressure, loss of body mass, and improved glucose tolerance [1-3]. This report concerns a serendipitous finding of a paradoxical increase in body mass and size in a pilot study with GPA, designed to prevent the development of hypertension in three-week-old, male spontaneously hypertensive rats (SHR).

## Materials and methods

Ethical approval and guidelines

The Animal Ethical Committee of the University of Amsterdam, the Netherlands, approved all the procedures described in this paper (Registry number DFC102100), which are in conformity with European legislation, and with the Federation of Laboratory Animal Science Associations (FELASA) recommendations, using the “3R” principle of Replacement, Reduction, and Refinement [6-7]. The ARRIVE (Animal Research: Reporting of in Vivo Experiments) guidelines were used to design and report the trial [8].

Key objective

To assess whether early inhibition of the intracellular CK system delays or reduces the development of hypertension in juvenile, pre-hypertensive SHR.

The spontaneously hypertensive rat

SHR, a genetic model of experimental hypertension engineered by inbreeding Wistar-Kyoto (WKY) rats, has similar blood pressure to WKY at three to five weeks of age, despite a generally lower body mass and size [3,9]. After five to six weeks, blood pressure rises in SHR, with major differences with WKY at nine weeks. At the early stage of hypertension, systemic vascular resistance is normal with increased cardiac output. In older rats, cardiac output may normalize or decrease while peripheral resistance rises. Typically, heart rate decreases with age in SHR [3,9]. High CK activities in SHR heart and aorta probably facilitate the development and sustenance of high blood pressure [3]. Early in life, at two days after birth, total cardiac CK/citrate synthase (CS) activity was higher in SHR (mU CK/U CS, male SHR n=6: 1377.7 (SE 113.8) vs WKY n=6: 993.2 (46.5), p<0.001; female SHR n=6: 1366.0 (40.9) vs WKY n=6: 1033.3 (59.2), p<0.01; unpublished results from convenience samples). In line with this finding, Jin et al. reported total cardiac CK around two-fold higher in four-week-old male SHR, preceding the development of hypertension. At 20 weeks, there was a four-fold increase in total CK in SHR vs WKY in this study, with blood pressure levels, respectively, 183.3 (7.5) in SHR and 135.0 (5.2) in WKY, which reduced with antihypertensive drugs [10]. Clark et al. also reported significantly higher cytoplasmic CK in 26-week-old male SHR, 0.203, SE 0.014 IU/mg soluble protein vs 0.159, SE 0:01 in WKY [11]. Finally, Seccia et al. found an increase in mitochondrial CK in male SHR by, respectively, 80% at five weeks, to 110% at 24 weeks compared to WKY, with a concurrent increase in SBP (week 5 to 24, respectively, in SHR 123 (SE 10) to 214 (14) vs to 115 (9) to 139 (12) in WKY) [12]. Hence, this animal model was suitable to assess whether early CK inhibition would prevent or reduce high blood pressure.

Creatine and GPA

The flux through the creatine kinase reaction is linearly dependent on the intracellular creatine concentration [13]. Creatine (NH_2_C(=NH)-N(CH_3_)-CH_2_-COOH) may be absorbed from animal-based dietary sources or synthesized de novo, which demands a substantial part of bioavailable L-arginine in a two-step, multi-organ process. L-arginine: glycine amidinotransferase (AGAT, EC 2.1.4.1) catalyzes the first, rate-limiting step of L-arginine and glycine to form guanidinoacetic acid (GAA) in the kidney. Secondly,* S*-adenosyl-L-methionine: *N*-guanidinoacetate methyltransferase (GAMT, EC 2.1.1.2), mainly in the liver, uses *S*-adenosyl-L-methionine to methylate GAA and produce creatine and *S*-adenosyl-L-homocysteine. Creatine is transported to plasma and enters tissues after passing the saturable, sodium- and chloride-dependent creatine transporter 1 (CT1) belonging to the solute carrier 6 family of transporters [1-3].

GPA (or *N*-(aminoiminomethyl)-beta-alanine; or 3-carbamimidamidopropanoic acid (NH_2_C(=NH)-NH-CH_^2^_-CH_2_-COOH) is a structural isomer and competitive inhibitor of creatine, probably synthesized by renal AGAT from L-arginine and beta-alanine [3,14]. GPA occurs in mammalian tissue, urine, and blood (estimated plasma concentration in healthy rats, 0.06, SD 0.02 μmol/L) and is cleared by the kidney [1-3]. In pharmacological doses, GPA reduces the flux through the CK reaction, mainly through competitive inhibition of creatine at CT1 (with a K_i _of 8.8 to 120 μmol/L) [2,3]. In addition, GPA is transported by CT1 to the cytoplasm as an ‘‘inefficient substrate’’ for the cytoplasmic CK reaction, acting as a competitive inhibitor [2-3]. GPA does not directly inhibit mitochondrial CK [2,3]. In animal studies, GPA 0.5% to 2% induced a shift toward oxidative metabolism, with greater endurance capacity, enhanced cellular glucose and fatty acid uptake, and loss of body mass or growth retardation [2]. Central to these changes is the activation of 5' adenosine monophosphate-activated protein kinase (AMPK, EC 2.7.11.31), increased mitochondrial activity, and an increase in GLUT4, probably induced by lowered cytoplasmic ATP [2]. In the unstressed heart of the intact animal, left ventricular systolic pressure and cardiac output were unchanged. Blood pressure reduced in adult SHR using GPA [3], and this study was designed to assess whether early CK inhibition in juvenile SHR could prevent or delay the development of hypertension.

Main intervention

In an attempt to find the lowest effective dose, GPA 0.1% added to nutritionally balanced, semi-synthetic plant (soy)-based (meat-free and creatine-free) chow with 20% soy protein, and 0.7% methionine (AB Animal Nutrition, Woerden, the Netherlands; Table 1.) vs control feed was used in three-week-old SHR during four (primary, t=4w) to 6 weeks (secondary, t=6w).

**Table 1:** Nutritional Profile Rodent Diet Standard vegetarian (creatine-free) chow for laboratory animals (AB Animal Nutrition, Woerden, the Netherlands)

Proximate Profile					Amino Acids			
Protein	%	18.1			Alanine	g/kg	7.12	
Fat	%	5.1			Arginine	g/kg	13.4	
Fibre	%	5.1			Aspartic acid	g/kg	20.7	
Minerals	%	2.1			Cystine	g/kg	2.3	
Moisture	%	7.0			Glutamic acid	g/kg	37.4	
Carbohydrate	%	57.7			Glycine	g/kg	7.3	
Caloric content	Kcal/g	3.63			Histidine	g/kg	4.5	
					Isoleucine	g/kg	8.5	
Ingredients					Leucine	g/kg	14.1	
Soy protein	%	20.0			Lysine	g/kg	11.0	
Dextrose	%	54.0			Methionine	g/kg	7,2	
Cornstarch	%	10.0			Phenylalanine	g/kg	9.4	
Soy oil	%	5.0			Proline	g/kg	9.5	
Cellulose	%	5.0			Serine	g/kg	9.0	
CaHPO_4_·2H_2_O	%	1.3			Threonine	g/kg	6.4	
CaCO_3_	%	1.0			Tryptophan	g/kg	2.6	
KH_2_PO_4 _	%	0.7			Tyrosine	g/kg	7.0	
KCl	%	0.7			Valine	g/kg	7.8	
DL-Methionine	%	0.5						
Choline chloride	%	0.4			Vitamins			
MgSO_4_·7H_2_O	%	0.4			Ascorbic acid	mg/kg	0.0	
NaCl	%	0.3			Biotin	mcg/kg	306.7	
Vitamin premix†	%	0.25			Choline	mg/kg	1736	
Trace el. premix	%	0.25			Folic acid	mg/kg	7.8	
MgO	%	0.2			Niacin	mg/kg	39.2	
					Pantothenic acid	mg/kg	15.9	
Minerals					Pyridoxine	mg/kg	15.3	
Calcium	g/kg	7.4			Riboflavin	mg/kg	11.6	
Chloride	g/kg	5.3			Thiamin	mg/kg	20.0	
Chromium	mg/kg	0.49			Vitamin A	IU/g	18.0	
Cobalt	mg/kg	0.14			Vitamin B12	mcg/kg	50	
Copper	mg/kg	16.8			Vitamin D3	IU/g	2.0	
Fluorine	mg/kg	2.1			Vitamin E	mg/kg	62.7	
Iodine	mg/kg	0.47			Vitamin K3	mg/kg	10.0	
Iron	mg/kg	125.9						
Magnesium	g/kg	1.6			Fatty acids			
Manganese	mg/kg	62.7			C8-C14:0	g/kg	<0.1	
Phosphorus	g/kg	5.6			C16:0 Palmitic	g/kg	5.0	
Potassium	g/kg	5.8			C16:1 Palmitoleic	g/kg	0.5	
Selenium	mg/kg	0.19			C18:0 Stearic	g/kg	2.0	
Sodium	g/kg	2.95			C18:1 Oleic	g/kg	11.0	
Sulfur	g/kg	0.5			C18:2 Linoleic	g/kg	27.5	
Zinc	mg/kg	48.6			C18:3 Linolenic	g/kg	3.78	

GPA was obtained from Purebulk Vitamins and Dietary Supplements (Roseburg, Oregon, USA). GPA purity was >99% by nuclear magnetic resonance analysis (VUMC, Division of Organic Chemistry, Department of Chemistry and Pharmaceutical Sciences, Amsterdam, the Netherlands). Cyanide, assessed because cyanamide is used in GPA synthesis, was below detection limits (<1 p.p.m.; Eurofins Omegam Laboratories, Amsterdam, the Netherlands).

Main outcome

The primary outcome was the difference in SBP at t=4w (secondary t=6w) between GPA and control. Other outcomes were body mass and contractility of isolated mesenteric resistance arteries.

Sample size calculation

Based on early antihypertensive treatment, a decrease of 15 mmHg (SD 10) (125 vs 110 mmHg) was expected [9], thus eight rats would be needed in each group (two-tailed alpha=0.05 and 1−beta=0.80).

Animals, housing, and husbandry

Animals were housed and cared for, as described previously [3], at the Animal Research Facility of the Academic Medical Center at the University of Amsterdam. Fourteen pregnant SHR were obtained from Charles River (Maastricht, the Netherlands; Originally from Okamoto, Kyoto School of Medicine, 1963, to National Institutes of Health, the USA in 1966 at F13, from there to Charles River in 1973 at F32; Coat Color White (albino); Strain Code 007). Dams received standard soy-based chow from the moment of arrival, around two weeks before delivery. At three-weeks-old, male SHR offspring were separated from dams and randomly (1:1) assigned to receive soy-based chow supplemented with GPA 0.1% vs control, stratified by dams. Animals were housed with two to three per cage with water and food ad libitum, using Lignocell S8/15 laboratory animal bedding (J. Rettenmaier & Söhne GmbH + Co. KG, Rosenberg, Germany) changed weekly. Ambient temperature was maintained between 19°C and 24°C, with a humidity of 40% to 60% (both checked and registered daily), and a light cycle of 12 h light (maximum 350 lux) and 12 h dark (7 pm to 7 am), in a sound-reduced and ultrasound-free environment. Pathogens were actively monitored and controlled [7]. Daily, food intake, and body mass were monitored, and animals were checked for behavioural, physical, or other health changes by the institute’s staff under the supervision of a clinical veterinarian.

Blood pressure measurement

Standardized tail-cuff blood pressure measurements of conscious rats were performed once weekly with the validated CODA system (Kent Scientific Corporation, Torrington, CT, USA), using a heating pad to obtain a body temperature of 35ºC to 37ºC. This device measures both SBP and diastolic blood pressure (DBP) using volume pressure-recording sensor technology [3]. During the measurements, which lasted 10 to 15 minutes, care was taken to minimize stress for the animals. The last five out of 10 measurements per cycle were used for the analyses.

Resistance artery analysis

Rats were sacrificed at t=4w (primary) or t=6w (secondary). Animals were anaesthetized by a certified staff member of the animal facility with ketamine (90 mg/kg)-dexmedetomidine (0.125 mg/kg)-atropine (0.05 mg/kg) (KMA) through intraperitoneal injection. Resistance artery analysis *ex vivo* was conducted as previously reported [1,3,15]. In brief, the intestine with its feeding vasculature was rapidly removed and immediately stored in cold (4°C) physiological salt solution (PSS) containing (in mM): NaCl 118.2, NaHCO_3_ 24.8, KCl 4.6, KH_2_PO_4_ 1.2, MgSO_4_ 1.2, CaCl_2_ 2, EDTA 0.026, HEPES 5, and glucose 5.5. Third-order resistance-sized mesenteric arteries (~200 to 400 μm normalized internal diameter) were carefully excised, cleaned from adherent adipose and connective tissue, cut into ~2-mm-long rings (2 to 4 per rat), and mounted on 40-μm stainless steel wires in a Mulvany-Halpern wire myograph (Danish Myo Technology, Copenhagen, Denmark). The myograph bath contained PSS at 37°C bubbled with 95% O_2_-5% CO_2_ mixture to achieve a pH of 7.4. The calibre of the vessel and settings for distension were calculated using standard methods for wire myography [1,3,15]. Vessels were included that were able to generate active tension against at least 50 mmHg equivalent pressures. Maximum contractility was induced in duplicate with norepinephrine (10^-5^mol/L) in KCl (125 mmol/L)-substituted physiologic salt solution (KPSS-NE) as previously described, with results standardized for vessel diameter and length [1,3,15]. After washing and 20 minutes of rest, viability was assessed. Vessels were pre-constricted with phenylephrine (10^-5^mol/L), adding methacholine (10^-6^mol/L) to assess whether endothelial-dependent vasodilation was intact. Hereafter, cumulative concentration-response curves for methacholine (10^-9^ to 10^-5 ^mol/L) and SNP (10^-9^ to 10^-4 ^mol/L) were constructed and the logarithm to the base of 10 of the concentration of that, which gives the half-maximal response, log_10_ (IC_50_), was calculated. All concentrations refer to final bath concentrations. Chemicals for the vascular studies were obtained from Sigma-Aldrich Chemical Co. (St. Louis, MO, USA).

Heart mass and heart-to-body mass ratio

Heart mass was determined after dissection and normalized to body mass.

Data analysis

The primary analysis was intent-to-treat, with GPA vs control rats as the primary unit of analysis. Parametric statistics were used for the primary analysis. Data were reanalyzed in a sensitivity analysis with non-parametric methods because of the small sample size. In addition, a multivariable regression analysis was conducted to assess predictors of blood pressure. Formal statistical testing was limited and one-sided p-values were only used with a hypothesis or evidence on the direction of the outcome present. The nature of missing data was analyzed. Data missing completely ad random, as assessed through inspection and with the Little’s test in SPSS, were imputed with unconditional means. Analyses were performed with IBM SPSS Statistics for Windows, Version 25.0. (IBM Corp. Armonk, NY), and GraphPad Prism version 9.0.0 for Windows (GraphPad Software, San Diego, CA). Data are presented as mean ± standard error (SE) unless indicated otherwise.

## Results

Animals

Twenty-two male SHR were available for this pilot study randomized to treatment with GPA 0.1% vs control creatine-free chow, stratified by dams where possible. Eleven rats were sacrificed at t=4w (5 GPA and 6 controls) and 11 at t=6w (6 GPA and 5 controls).

Body mass, food intake, and general health

**Figure 1:**
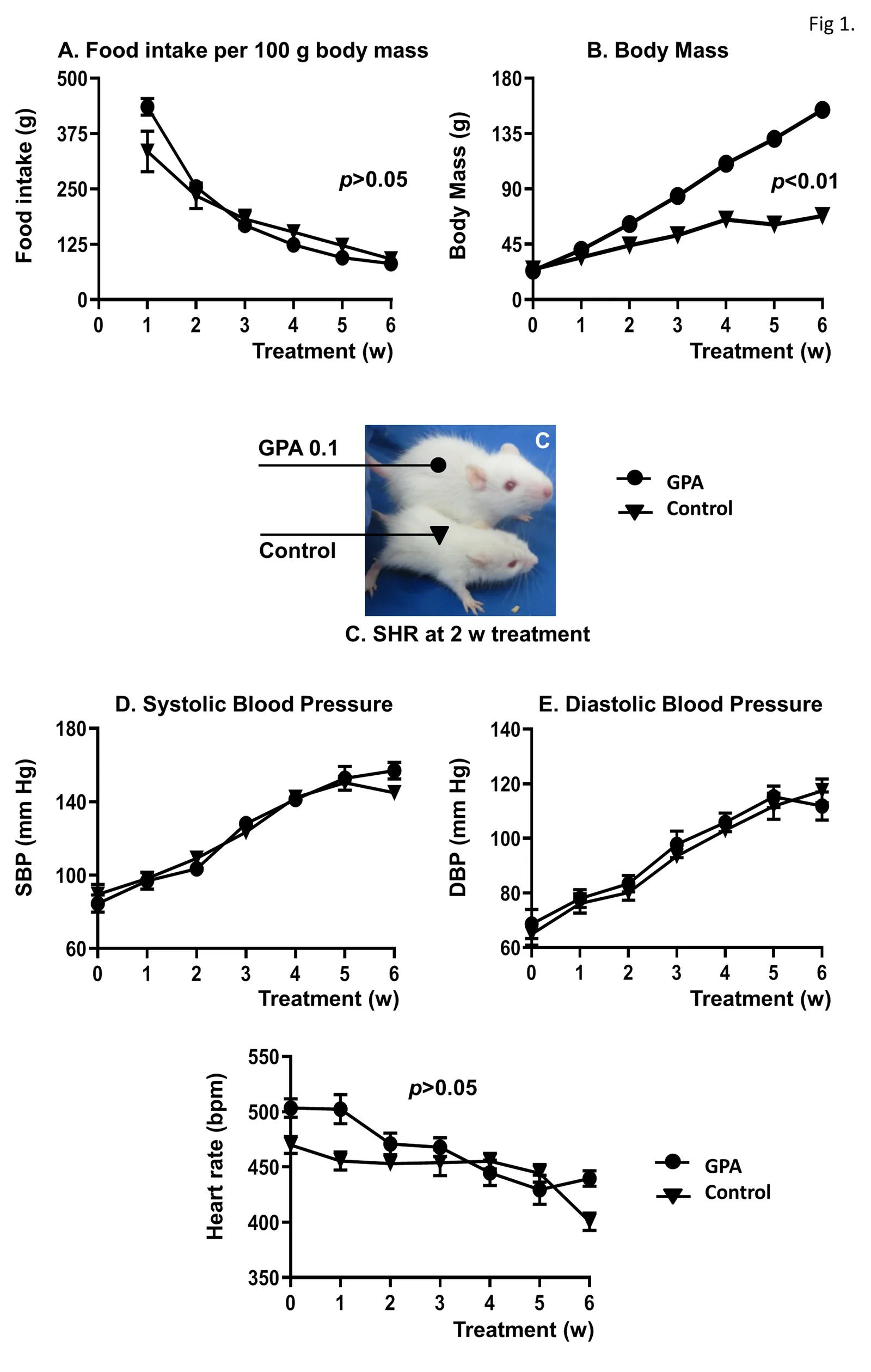
Food intake and clinical parameters GPA vs control chow (Panels A to F) Mean food intake per week in grams (g), per 100 g body mass (Panel A); and body mass (g) (Panel B), of three-week-old spontaneously hypertensive rats (SHR) treated with beta-guanidinopropionic acid (GPA) 0.1% vs control standard creatine-free chow during four to six weeks (w) of treatment, showing a large increase in body mass with GPA despite similar food intake per 100 g body mass. Baseline (3 weeks of age) through t=4w (7 weeks of age), n=22; and t=6w (9 weeks of age), n=11 (rats were sacrificed at t=4w (n=11) or t=6w (n=11) for cardiovascular assessments). Panel C shows representative SHR on GPA vs control at t=2w (5 weeks of age), when differences in body mass and size were already apparent. Systolic blood pressure (SBP) and diastolic blood pressure (DBP), and heart rate (Panel D to F) were not significantly different between groups throughout the trial. Error bars are SE.

Body mass at three weeks of age, at baseline, was similar for GPA (n=11) vs control (n=11), 23.9 (0.8) vs 24.5 (0.8), respectively. Food intake per 100-gram animal mass was similar in both groups (Figure 1, Panel A.). Surprisingly, after one week of treatment (4 weeks of age), body mass was significantly higher in the GPA group (n=11) vs control (n=11) 40.0 (1.5) vs 32.1 (1.2) as the most striking observation, with a concurrent increase in body size, and this difference increased during the next weeks (Figure 1, Panels B and C). At t=4w (seven weeks of age), body mass (g) in GPA (n=11) vs control (n=11) was 110.4 (3.7) vs 65.0 (4.8), (+69.8%; p<0.001). When clustered by litter (if the litter was large enough to have 2 or more animals randomized to GPA and control arms), differences were similar, with a mean of 83.9% higher body mass at t=4w in the GPA arm vs controls of the same litter (n=4 litters with 18 rats, 9 in the GPA and 9 in the control group). As a post-hoc measure, chow and GPA were reanalyzed. These analyses confirmed that the rats with a “growth spurt” received 0.1% GPA. Animals appeared healthy and displayed normal physical activity throughout the study, despite the differences in body mass and size.

Blood pressure and heart rate

Systolic blood pressure (SBP) and diastolic blood pressure (DBP) increased, and heart rate decreased with age, but without a significant difference between groups despite the large difference in body mass and body size (Figure 1, Panels D to F). SBP at t=4w was analyzed with linear multivariable regression analysis using body mass and intervention status as predictors, SBP=132.39 + 0.15* body mass -7.93*GPA, underlining the relatively low blood pressure in GPA-treated SHR.

Heart mass and heart to body mass ratio

At t=4w, in line with the larger body size, mean heart mass ex vivo was higher in the GPA group (0.97 (0.15) g vs control 0.50 (0.04) g, p<0.01); but mean heart mass/body mass ratio was not significantly different at this sample size (GPA 8.9 (1.4) mg/g vs control 6.8 (0.7), p=0.39).

Myograph experiments

**Figure 2:**
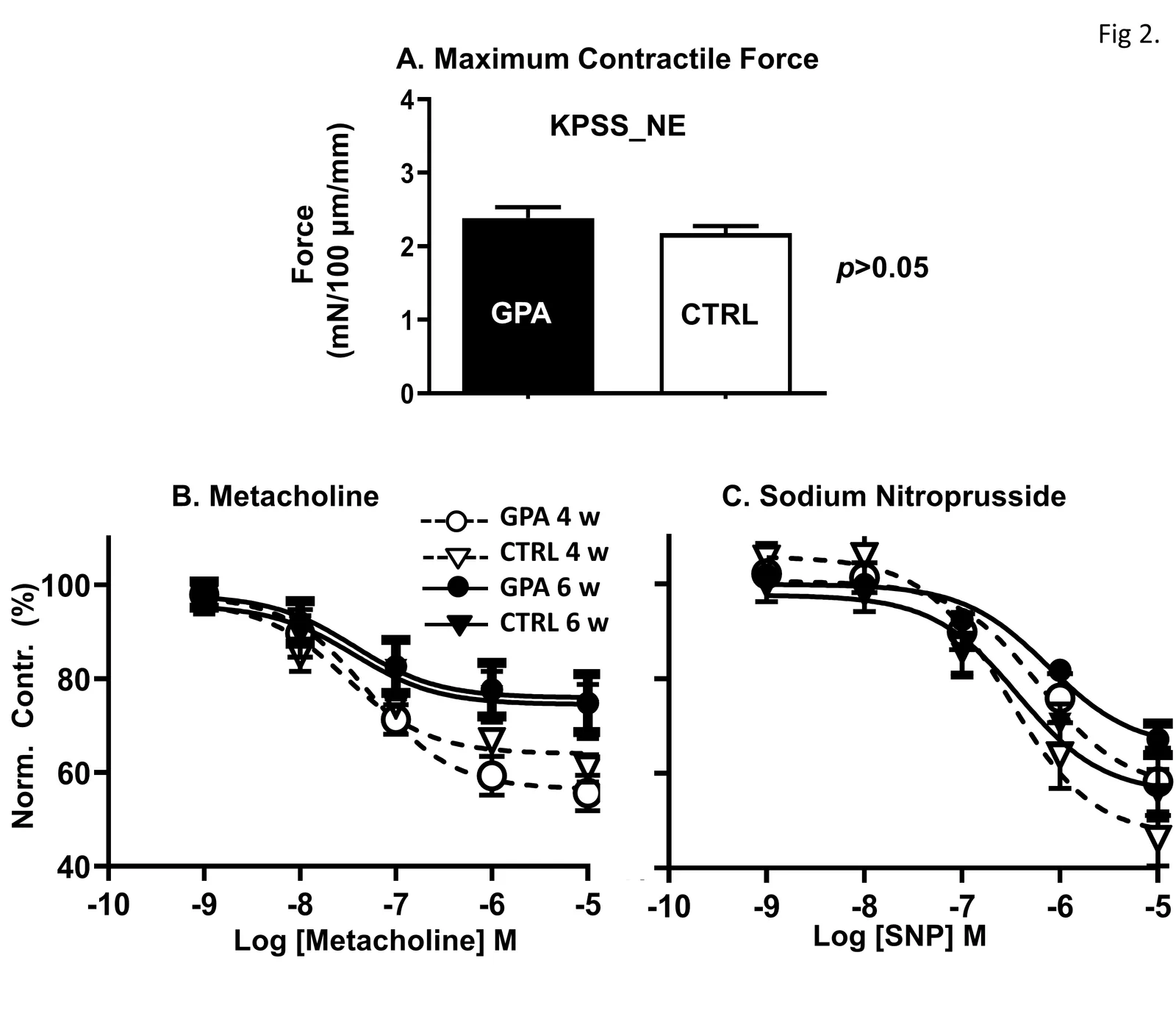
Resistance artery contractility GPA vs control (Panels A to C) Contractility responses in isolated mesenteric resistance-sized arteries (n=2 to 4 per animal) of beta-guanidinopropionic acid (GPA)-treated (n=11) vs control (CTRL) (n=11) spontaneously hypertensive rats (SHR). Vasoconstriction induced with KPSS-NE (expressed as force in mN per 100 μm vessel diameter per mm artery length) was not significantly different between SHR and CTRL (pooled data of t=4 weeks (w) and t=6w; n=11 in each group; panel A). Vasodilation at t=4w (GPA n=5 vs CTRL n=6) and t=6w (GPA n=6 vs CTRL n=5) was induced after pre-constriction with KPSS-NE, with cumulative concentrations of methacholine (Panel B) or sodium nitroprusside (SNP, Panel C). Data are expressed as percentage vasodilation of the maximum contractile response. Error bars are SE. Residual contractility after methacholine was significantly higher at t=6w than at t=4w in both groups, but contractility characteristics were not significantly different for GPA vs CTRL. Norm. contr., normalized contractility.

From each rat in the control (n=11) and GPA groups (n=11), two to four (median 2) mesenteric arteries were assessed. Results of multiple vessels per rat were averaged. The mean normalized vessel diameter was 248.1 μm (13.3) in the GPA group (n=11) vs 196.8 (9.6) in controls (n=11), p<0.01. Maximum contractile force to KPSS-NE did not differ significantly between groups, GPA (n=11) 2.38 (0.15) mN/mm vessel length/100 µm vessel diameter vs 2.18 (0.10) in controls (n=11), (Figure 2, Panel A). Reduction in normalized contraction as assessed by methacholine (10^-9 ^to 10^-5^) showed no significant difference by intervention (GPA vs control), but residual vasoconstriction was significantly higher at t=6w than at t=4w, as reported for SHR [3,9] (Figure 2, Panel B; Table 2). However, Log IC_50_ was the same order of magnitude at t=4w and 6w in both groups (Table 2). Vascular dilation with SNP was higher than for methacholine as expected [1,3,15] but not different between treatment groups (Figure 2, Panel C; Table 2).

**Table 2:** Isolated resistance artery parameters GPA vs control Characteristics of isolated mesenteric resistance-sized arteries (n=2 to 4 per animal) ex vivo after beta-guanidinopropionic acid (GPA) vs control (CTRL) in spontaneously hypertensive rats. Vasoconstriction induced with KPSS-NE is expressed as force in mN per 100 μm vessel diameter and per mm artery length. Vasodilation induced with cumulative concentrations of methacholine (MCH) or sodium nitroprusside (SNP) after preconstruction with KPSS-NE is expressed as percentage vasodilation of the maximum contractile response, indicating significantly greater endothelial-dependent vasodilation at t=4 weeks (w) vs t=6w in both GPA-treated and CTRL rats. There were no significant differences between GPA and CTRL, despite significantly larger artery diameter in GPA-treated rats. Contr., contractility. IC 50, half-maximal inhibitory concentration.

	4 weeks		6 weeks		
	GPA (n=5)	CTRL (n=6)	GPA(n=6)	CTRL (n=5)	p<0.05
Artery diameter, μm	213.2 (7.2)^a^	197.9 (14.3)^b^	277.2 (15.6)^c^	195.4 (13.7)^d^	a vs b; a vs c
Force (mN/100μm/mm)	2.22 (0.16)	2.15 (0.15)	2.51 (0.24)	2.20 (0.14)	ns
MCH, residual contr., %	55.7% (3.8)^a^	60.9 (2.9)^b^	74.8% (6.2)^c^	73.0 (5.8)^d^	a vs c; b vs d
MCH, Log IC 50	−7.28 (0.21)	−7.52 (0.21)	−7.43 (0.56)	−7.44 (0.57)	ns
SNP, residual contr., %	58.2 (7.1)	46.2 (5.7)	67.2 (3.3)	55.5 (5.3)	ns
SNP, Log IC 50	−6.21 (0.26)	−6.12 (0.24)	−6.44 (0.15)	−6.44 (0.24)	ns

Missing data and sensitivity analysis

The follow-up for the primary outcome was complete. There were no dropouts, and all animals could be analyzed for the primary outcome with an intent-to-treat analysis. Data for SNP, missing for one rat on GPA and one control at t=4w, and for one concentration (SNP 10^-9^) in two GPA-treated rats t=6w, were considered missing ad random and were imputed. Data reanalyzed without imputation or with non-parametric methods showed no change in the magnitude or direction of outcomes.

## Discussion

Enhanced growth in SHR fed GPA

The aim of this study was to prevent or reduce hypertension in three-week-old SHR with GPA, a competitive inhibitor of CT1 and cytoplasmic CK that reduces intracellular ATP [1-3]. High CK in SHR is thought to facilitate hypertension through greater availability of ATP [3,10-12]. With the CK reaction highly dependent on creatine [1,13], standard vegetarian rat chow was used in this trial to limit creatine availability to endogenous synthesis. To our knowledge, GPA was not given to young, pre-hypertensive SHR on a creatine-free diet previously, and the trial yielded unexpected findings [1,2].

Within a week, rats on GPA were noticed to develop a greater body size and mass than controls. SHR is a smaller rat than the WKY rat [3,9,16]. In one report, the body mass of three-week-old SHR weanlings grew from 28.4 (0.71) g to 103.7 (2.5) in six-week-old SHR, vs 32.9 (1.3) to 116.5 (3.3) in WKY [16]. In this study on SHR, the mean body mass of three-week-old weanlings at baseline was 24.2 (0.5) g to 84.3 (2.6) g at six weeks of age in GPA-treated rats vs 52.3 (3.5) in controls. Thus, both GPA and control were probably smaller than usual for SHR, but the GPA-fed rats grew closer to the historical values for SHR body mass than controls. Despite the significantly higher body mass in GPA-treated SHR, there were no differences in systolic or diastolic blood pressure or other cardiovascular characteristics vs controls [3,9]. Because of growth retardation reported with GPA in young mammals [2], the chow and GPA were reanalyzed but no abnormalities were found. Furthermore, using the same GPA batch, blood pressure was successfully lowered in studies in 16-week- old SHR with GPA (baseline SBP 191.5 (4.3) reduced by 42.7 (5.5) mmHg compared to controls) [3]. Also, a first-in-human tolerance study with GPA was uneventful [1].

Limitations

**Figure 3:**
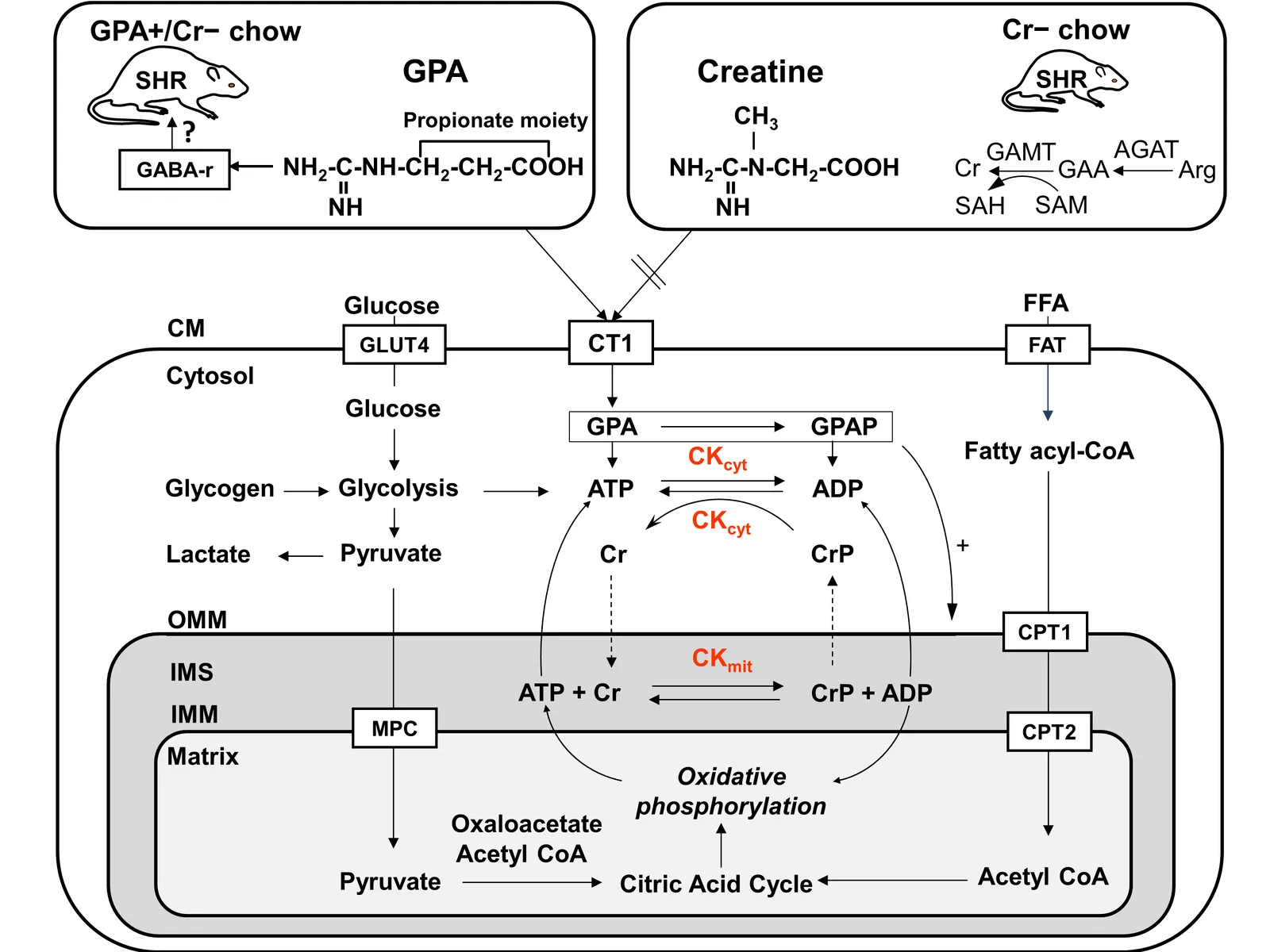
Potential mechanism of increased body mass with GPA Unexpected growth in three-week-old spontaneously hypertensive rats (SHR) randomized to four to six weeks beta-guanidinopropionic acid (GPA) 0.1% vs control, standard creatine-free chow. Arginine: glycine amidinotransferase (AGAT) catalyzes the first step of endogenic creatine synthesis, to guanidinoacetate (GAA) from arginine (and glycine). Subsequently,* S*-adenosyl-methionine: guanidinoacetate methyl-transferase (GAMT) catalyzes the methyl transfer from *S*-adenosyl-L-methionine (SAM) to GA, yielding *S*-adenosyl-L-homocysteine (SAH) and creatine (Cr), the substrate of the creatine kinase (CK) reaction. CK is a central regulatory enzyme of cellular energy metabolism. Mitochondrial CK (CKmit) located in the intermembraneous space (IMS) between the outer (OMM) and inner mitochondrial membrane (IMM) synthesizes creatine phosphate (CrP) from adenosine triphosphate (ATP) generated in the matrix, creating a phosphoryl group shuttle toward the cytoplasm. Cytosolic CK (CKcyt) utilizes CrP to rapidly provide ATP to ATPases such as Na^+^/K^+^-, Ca^2+^-, and myosin ATPase. Thus, CK promotes sodium retention, vascular contractility, and pressor responses. The flux through the CK reaction is highly dependent on intracellular (creatine). GPA competitively inhibits cellular creatine uptake at the creatine transporter 1 (CT1) in the cellular membrane (CM), resulting in an attenuated flux through the CK reaction. GPA is also competitive at CKcyt and is phosphorylated by this enzyme. GPA does not directly inhibit CKmit. Lower ATP/ADP ratios with GPA stimulate mitochondrial respiration and biogenesis, enhance glucose transporter protein-4 (GLUT4) expression, glucose uptake, fatty acid transporter (FAT) expression, and free fatty acid (FFA) uptake [1-3]. Mitochondrial stimulation, partial creatine or GABA agonist effects, or potential effects of propionate could have contributed to enhance growth in juvenile SHR. CPT, carnitine palmitoyltransferase; MPC, mitochondrial pyruvate carrier.

The main limitation is that this study was not designed to address growth responses. Ethical permission was obtained for the dose-finding study with GPA to reduce blood pressure in juvenile SHR, which was executed as planned. The new findings are reported as observed, a relatively small body size with creatine-free food and the “catch-up” growth with GPA, in rats with relatively high CK. Our current or historic data on GPA use in mammals [2-3] provided no data to explain the unexpected growth with GPA and thus the mechanisms by which these alterations take place remain obscure. Nevertheless, some explanatory pathways are suggested (Figure 3).

Hypotheses

Deficient Diet: Methionine, Creatine, or Calorie Lack

A plant-based diet is almost universally regarded as better for cardiovascular health, albeit with potential growth retardation in young animals [17-18]. Plant-based diets are creatine-free, and within the context of a high creatine demand (because of high CK) [13], SHR had to synthesize creatine de novo from arginine and lysine, needing methionine, an essential amino acid involved in cysteine and glutathione synthesis, and the primary methyl donor in creatine synthesis [1,19]. Rat embryos are able to synthesize creatine, and plasma creatine is reported to be normal in rats on a vegetarian diet, albeit with higher levels of creatine supplementation [20]. On the other hand, more recent reports indicate that a soy-based (vegetarian) diet compared to a casein or meat-based diet given to rats was associated with significantly reduced daily feed intake (decreased by 9%, p< 0.05), reduced body mass gain (decreased by 65%, p< 0.05); and lower methionine levels, with greater urea production, potentially related to increased deamination of non-essential amino acids [21]. However, the soy-based chow given to SHR was methionine-supplemented, and rats on SHR vs control had similar food intake per 100 g body mass. Possibly, the available methionine did not meet the demand for higher creatine synthesis in SHR, and this might have contributed to lower body mass compared to historical SHR data, but this does not explain the higher body mass with GPA vs control.

GPA as Substitute Creatine

Could GPA have acted as a “partial creatine agonist”? GPA is reported to lead to growth retardation in young rats on standard chow, but young rats with relatively high CK or on a creatine-free diet were not previously studied [2]. In this study, GPA was used at a rather low dose, 0.1% vs up to 4% in other studies [2]. GPA has a high affinity for creatine-binding places, including at CT1 and CKcyt, where it acts as a competitive inhibitor in the presence of creatine but is also reported to be transported into the cell by CT1 and phosphorylated and used by CK [2,22]. It is speculated that in the young, high-CK SHR on a creatine-free diet, where endogenous agonist creatine was low and demand was high [1,3], GPA might have acted as substitute creatine, thus as a partial agonist instead of a competitive inhibitor [2,22-23].

GPA-Induced Mitochondrial Activity

Because of lower intracellular ATP, GPA is known to induce a switch from anaerobic to aerobic metabolism and activate AMPK, a highly conserved sensor of low intracellular ATP levels. This leads to enhanced mitochondrial respiration and biogenesis [2]. Although this metabolic switch is reported to result in loss of body mass in adult and juvenile mammals [2], at very low creatine levels and higher demands in juvenile SHR, this might have contributed to enhanced growth.

Non-CK System GPA Effects

Related to its structural similarity with gamma-aminobutyric acid (GABA, NH_2_-CH_2_-CH_2_-CH_2_-COOH), GPA is reported to have a high affinity for the GABA_A_ receptor, where it may act as a partial agonist [24]. GABA may increase energy intake, GAA uptake in the liver, creatine synthesis, and growth hormone levels [25-27]. Importantly, GABA signalling is immature in the young rat, and the effect may be stimulatory rather than inhibitory [25]. Thus, the effect of GPA on GABA-ergic signalling, if any, would depend on the age of the animal, the concentration of the endogenous agonist, and the degree of constitutive GABA receptor activity [23]. It is unknown whether the propionate moiety in GPA could have contributed to the observed effects [28]. Dedicated replication studies, preferably with alternatives for animal testing [29], will be needed to find the cause of the observed effects of GPA on growth.

## Conclusions

Perspective

Hypertension remains a major risk factor for premature death worldwide, and there is an urgent need for preventive strategies applicable to young, pre-hypertensive populations to reduce early mortality. This pilot study was designed to assess whether early CK inhibition with the creatine analogue GPA could slow down or prevent the development of hypertension in juvenile, three-week-old SHR on endogenous creatine synthesis. CK, a central regulatory enzyme energy metabolism, is intimately involved in blood pressure generation through rapid ATP regeneration from phosphocreatine at subcellular ATPases that execute pressor responses. GPA, a competitive inhibitor of creatine at the transporter CT1 and at cytoplasmic CK, lowers intracellular ATP, blood pressure, and body mass but potentially induces growth retardation in young mammals. Surprisingly, three-week-old SHR given GPA 0.1% added to soy-based chow vs controls displayed a significant increase in body size and body mass (+70%) compared to controls. Cardiovascular parameters, including blood pressure, were not different between groups. Some explanatory pathways are suggested, including a potential (partial) agonist effect of GPA related to low levels of the endogenous agonist creatine. Replication studies, preferably using alternatives to animal testing, are needed to confirm these findings and to further understand the regulation of energy metabolism in young, hypertension-prone mammals on a vegetarian diet.

## References

[1] Brewster LM (2018). Creatine kinase, energy reserve, and hypertension: from bench to bedside. Ann Transl Med.

[2] Oudman I, Clark JF, Brewster LM (2013). The effect of the creatine analogue beta-guanidinopropionic acid on energy metabolism: a systematic review. PLoS One.

[3] Karamat FA, Oudman I, Haan YC (2016). Creatine kinase inhibition lowers systemic arterial blood pressure in spontaneously hypertensive rats. A randomized controlled trial. J Hypertens.

[4] Brewster LM, Haan YC, Zwinderman AH, van den Born BJ, van Montfrans GA (2020). Ck (creatine kinase) is associated with cardiovascular hemodynamics. The HELIUS Study. Hypertension.

[5] Brewster LM, Mairuhu G, Bindraban NR, Koopmans RP, Clark JF, van Montfrans GA (2006). Creatine kinase activity is associated with blood pressure. Circulation.

[6] (2021). European Parliament. The European Union Directive 2010/63/EU revising Directive 86/609/EEC on the protection of animals used for scientific purposes. http://eur-lex.europa.eu/legal-content/EN/TXT/PDF/?uri=CELEX:32010L0063&from=EN.

[7] Mähler Convenor M, Berard M, Feinstein R, Gallagher A, Illgen-Wilcke B, Pritchett-Corning K, Raspa M (2014). FELASA recommendations for the health monitoring of mouse, rat, hamster, guinea pig and rabbit colonies in breeding and experimental units. Lab Anim.

[8] Kilkenny C, Browne WJ, Cuthill IC, Emerson M, Altman DG (2010). Improving bioscience research reporting: the ARRIVE guidelines for reporting animal research. PLoS Biol.

[9] Dickhout JG, Lee RM (1998). Blood pressure and heart rate development in young spontaneously hypertensive rats. Am J Physiol.

[10] Jin X, Xia L, Wang LS (2006). Differential protein expression in hypertrophic heart with and without hypertension in spontaneously hypertensive rats. Proteomics.

[11] Clark JF, Radda GK, Boehm EA (2000). The effects of anti-hypertensive therapy on the structural, mechanical and metabolic properties of the rat aorta. J Muscle Res Cell Motil.

[12] Seccia TM, Atlante A, Vulpis V, Marra E, Passarella S, Pirrelli A (1998). Mitochondrial energy metabolism in the left ventricular tissue of spontaneously hypertensive rats: abnormalities in both adeninenucleotide and phosphate translocators and enzyme adenylate-kinase and creatine-phosphokinase activities. Clin Exp Hypertens.

[13] Meyer RA (1989). Linear dependence of muscle phosphocreatine kinetics on total creatine content. Am J Physiol.

[14] Fritsche E, Humm A, Huber R (1999). The ligand-induced structural changes of human L-arginine:glycine amidinotransferase. A mutational and crystallographic study. J Biol Chem.

[15] Taherzadeh Z, Karamat FA, Ankum WM, Clark JF, van Montfrans GA, van Bavel E, Brewster LM (2016). The effect of creatine kinase inhibition on contractile properties of human resistance arteries. Am J Hypertens.

[16] Gattone VH II (1986). Body weight of the spontaneously hypertensive rat during the suckling and weanling periods. Jpn Heart J.

[17] Giuberti G, Morlacchini M, Crippa L, Capraro J, Paganini B, Gallo A, Rossi F (2018). Effect of omnivorous and vegan diets with different protein and carbohydrate content on growth and metabolism of growing rats. Int J Food Sci Nutr.

[18] Herring CM, Bazer FW, Johnson GA, Wu G (2018). Impacts of maternal dietary protein intake on fetal survival, growth, and development. Exp Biol Med (Maywood).

[19] Braissant O, Henry H, Villard AM, Speer O, Wallimann T, Bachmann C (2005). Creatine synthesis and transport during rat embryogenesis: spatiotemporal expression of AGAT, GAMT and CT1. BMC Dev Biol.

[20] Brault JJ, Abraham KA, Terjung RL (2003). Muscle creatine uptake and creatine transporter expression in response to creatine supplementation and depletion. J Appl Physiol (1985).

[21] Song S, Hooiveld GJ, Li M (2016). Dietary soy and meat proteins induce distinct physiological and gene expression changes in rats. Sci Rep.

[22] Fitch CD, Jellinek M, Fitts RH, Baldwin KM, Holloszy JO (1975). Phosphorylated beta-guanidinopropionate as a substitute for phosphocreatine in rat muscle. Am J Physiol.

[23] Berg KA, Clarke WP (2018). Making sense of pharmacology: inverse agonism and functional selectivity. Int J Neuropsychopharmacol.

[24] Feltz A (1971). Competitive interaction of beta-guanidino propionic acid and gamma-aminobutyric acid on the muscle fibre of the crayfish. J Physiol.

[25] Maejima Y, Yokota S, Horita S, Shimomura K (2019). The undeveloped properties of GABA neurons in the ventral tegmental area promote energy intake for growth in juvenile rats. Sci Rep.

[26] Tachikawa M, Ikeda S, Fujinawa J, Hirose S, Akanuma S, Hosoya K (2012). γ-Aminobutyric acid transporter 2 mediates the hepatic uptake of guanidinoacetate, the creatine biosynthetic precursor, in rats. PLoS One.

[27] Oketch-Rabah HA, Madden EF, Roe AL, Betz JM (2021). United States Pharmacopeia (USP) safety review of gamma-aminobutyric acid (GABA). Nutrients.

[28] Tirosh A, Calay ES, Tuncman G (2019). The short-chain fatty acid propionate increases glucagon and FABP4 production, impairing insulin action in mice and humans. Sci Transl Med.

[29] Gygera M (2012). Should we thank our laboratory animals for giving their life for science?. Bioethica Forum.

