# Correction: Paradoxical Increase in Body Mass Induced by Beta-Guanidinopropionic Acid in Juvenile Spontaneously Hypertensive Rats

**DOI:** 10.7759/cureus.c494

**Published:** 2026-10-21

**Authors:** L M Brewster

**Affiliations:** 1 Cardiovascular Disease and Cardiovascular Population Health, Creatine Kinase Foundation, Amsterdam, NLD

Paragraph 1 of the Introduction section has been corrected to state that the enzyme code of creatine kinase is EC.2.7.3.2. This was previously misstated as EC 2.7.3.1.

